# Supplementary material for: Cortical encoding of acoustic and linguistic rhythms in spoken narratives
Source: eLife. 2020 Dec 21;9:e60433. doi: 10.7554/eLife.60433 (PMC7775109; doi:10.7554/eLife.60433)
Supplement: Supplementary file 1. [file elife-60433-supp1.docx]

***Assessment of the stimulus***

Question 1: Do you think there is noticeable difference between metrical and nonmetrical stories except for their content?

| Choice | *N* (%) |
| --- | --- |
| Have a noticeable difference. | 3 (9%) |
| Have no difference. | 31 (91%) |

Question 2: Do you think the two stories are naturally read?

| Choice | *N* (%) |
| --- | --- |
| Both stories are naturally read. | 24 (71%) |
| The metrical story is not naturally read. | 3 (9%) |
| The nonmetrical story is not naturally read. | 0 (0%) |
| Neither of the two stories is naturally read. | 7 (20%) |

Question 3: Which speech sounds more natural and is easier to understand?

| Choice | *N* (%) |
| --- | --- |
| σ1-amplified speech | 15 (44%) |
| σ2-amplified speech | 2 (6%) |
| Neither speech | 17 (50%) |
