# Supplementary material for: Cortical encoding of acoustic and linguistic rhythms in spoken narratives
Source: eLife. 2020 Dec 21;9:e60433. doi: 10.7554/eLife.60433 (PMC7775109; doi:10.7554/eLife.60433)
Supplement: Supplementary file 2. [file elife-60433-supp2.docx]

***Post-hoc effect size calculation***

***Experiment 1***

Effect size of the 2-Hz response for metrical stories

| Condition | *p-value* | *d* | power |
| --- | --- | --- | --- |
| isochronous speech | 0.0016 | 1.21 | 0.99 |
| σ1-amplified speech | 0.0009 | 1.15 | 0.99 |
| σ2-amplified speech | 0.0007 | 1.16 | 0.99 |

***Experiment 2***

Effect size of the 2-Hz response for metrical stories

| Condition | *p-value* | *d* | power |
| --- | --- | --- | --- |
| σ1-amplified speech | 0.0026 | 0.97 | 0.95 |
| σ2-amplified speech | 0.0021 | 1.04 | 0.97 |

***Experiment 3***

Effect size of the 2-Hz response for metrical stories

| condition | *p-value* | *d* | power |
| --- | --- | --- | --- |
| natural speech | 0.0109 | 0.76 | 0.81 |

***Experiment 4***

Effect size of the 2-Hz response for metrical stories

| Condition | *p-value* | *d* | power |
| --- | --- | --- | --- |
| σ1-amplified speech (story) | 0.0005 | 1.19 | 0.99 |
| σ2-amplified speech (story) | 0.0012 | 0.89 | 0.98 |
| σ1-amplified speech (movie) | 0.012 | 0.65 | 0.88 |
| σ2-amplified speech (movie) | 0.0031 | 0.80 | 0.97 |
