# Supplementary material for: Cortical encoding of acoustic and linguistic rhythms in spoken narratives
Source: eLife. 2020 Dec 21;9:e60433. doi: 10.7554/eLife.60433 (PMC7775109; doi:10.7554/eLife.60433)
Supplement: Transparent reporting form [file elife-60433-transrepform.docx]

***eLife’s* transparent reporting form**

We encourage authors to provide detailed information *within their submission* to facilitate the interpretation and replication of experiments. Authors can upload supporting documentation to indicate the use of appropriate reporting guidelines for health-related research (see [EQUATOR Network](http://www.equator-network.org/%20)), life science research (see the [BioSharing Information Resource](https://biosharing.org/)), or the [ARRIVE guidelines](http://www.plosbiology.org/article/info:doi/10.1371/journal.pbio.1000412) for reporting work involving animal research. Where applicable, authors should refer to any relevant reporting standards documents in this form.

If you have any questions, please consult our Journal Policies and/or contact us: [editorial@elifesciences.org](mailto:editorial@elifesciences.org).

**Sample-size estimation**

- You should state whether an appropriate sample size was computed when the study was being designed
- You should state the statistical method of sample size computation and any required assumptions
- If no explicit power analysis was used, you should describe how you decided what sample (replicate) size (number) to use

Please outline where this information can be found within the submission (e.g., sections or figure legends), or explain why this information doesn’t apply to your submission:

We applied a post-hoc power analysis, shown in Supplementary Table 2. The calculation method was described in *Materials and methods* (*Post-hoc effect size calculation*). We also showed the results from individual participants and individual EEG electrodes in supplementary Figure 2 - figure supplement 1 and Figure3 - figure supplement 1.

Furthermore, the current study relied on a neural marker established in previous studies, i.e., a 2-Hz neural response entrained to linguistic units, and the sample size was chosen to be slightly larger than previous studies (16 participants in the current study, compared to 8 participants for each MEG experiment in Ding et al., Nature Neurosci 2016 and 14 participants for each EEG experiment in Ding et al., J Neurosci 2018).

**Replicates**

- You should report how often each experiment was performed
- You should include a definition of biological versus technical replication
- The data obtained should be provided and sufficient information should be provided to indicate the number of independent biological and/or technical replicates
- If you encountered any outliers, you should describe how these were handled
- Criteria for exclusion/inclusion of data should be clearly stated
- High-throughput sequence data should be uploaded before submission, with a private link for reviewers provided (these are available from both GEO and ArrayExpress)

Please outline where this information can be found within the submission (e.g., sections or figure legends), or explain why this information doesn’t apply to your submission:

102 participants took part in the study. The study consisted of 1 behavioral test and 4 EEG experiments. Behavioral test involved 34 participants. EEG Experiments 1-3 involved 16 participants respectively, and EEG Experiment 4 involved 20 participants. All participants were included in the analysis. The details were described in *Materials and methods*.

**Statistical reporting**

- Statistical analysis methods should be described and justified
- Raw data should be presented in figures whenever informative to do so (typically when N per group is less than 10)
- For each experiment, you should identify the statistical tests used, exact values of N, definitions of center, methods of multiple test correction, and dispersion and precision measures (e.g., mean, median, SD, SEM, confidence intervals; and, for the major substantive results, a measure of effect size (e.g., Pearson's r, Cohen's d)
- Report exact p-values wherever possible alongside the summary statistics and 95% confidence intervals. These should be reported for all key questions and not only when the p-value is less than 0.05.

Please outline where this information can be found within the submission (e.g., sections or figure legends), or explain why this information doesn’t apply to your submission:

Statistical analysis methods were described in *Statistical tests* section. The exact p-values were reported in *Results*. The SEM were shown in response spectrums (Figure 2, Figure 4, Figure 5, and Figure 5 - figure supplement 1) and ERPs (Figure 6). The 95% confidence intervals were shown in individual results (Figure 2 - figure supplement 1, and Figure 3 – figure supplement 1).

(For large datasets, or papers with a very large number of statistical tests, you may upload a single table file with tests, Ns, etc., with reference to sections in the manuscript.)

**Group allocation**

- Indicate how samples were allocated into experimental groups (in the case of clinical studies, please specify allocation to treatment method); if randomization was used, please also state if restricted randomization was applied
- Indicate if masking was used during group allocation, data collection and/or data analysis

Please outline where this information can be found within the submission (e.g., sections or figure legends), or explain why this information doesn’t apply to your submission:

The study consisted of 1 behavioral test and 4 EEG experiments. 102 participants were randomly assigned to the behavioral test and EEG experiments. Behavioral test involved 32 participants, EEG Experiments 1-3 involved 16 participants respectively, and EEG Experiment 4 involved 20 participants. Auditory stimuli were presented in a randomized order in each Experiment (described in *Materials and methods*).

**Additional data files (“source data”)**

- We encourage you to upload relevant additional data files, such as numerical data that are represented as a graph in a figure, or as a summary table
- Where provided, these should be in the most useful format, and they can be uploaded as “Source data” files linked to a main figure or table
- Include model definition files including the full list of parameters used
- Include code used for data analysis (e.g., R, MatLab)
- Avoid stating that data files are “available upon request”

Please indicate the figures or tables for which source data files have been provided:

The EEG data and analysis code (in MATLAB) were uploaded as *Source Data* and *Source Code File*.
